# Heterogeneity in Self-Reported Cognitive Impairment Life Expectancies Among Older Latino Subgroups in the United States

**DOI:** 10.1093/geroni/igaa057.2076

**Published:** 2020-12-16

**Authors:** Marc Garcia, Kasim Ortiz, Chi-Tsun Chui, Brian Downer, David Warner

**Affiliations:** 1 University of Nebraska, Lincoln, Nebraska, United States; 2 University of New Mexico, Albuquerque, New Mexico, United States; 3 Academia Sinica, Taiwan, Taipei, Taiwan (Republic of China); 4 The University of Texas Medical Branch at Galveston, Galveston, Texas, United States; 5 University of Alabama at Birmingham, Birmingham, Alabama, United States

## Abstract

Research suggests the prevalence of cognitive impairment among older U.S. Latinos is increasing relative to the older U.S. population which has experienced and overall decline. This has been attributed in part to extended longevity, lower educational attainment, and a higher prevalence of diabetes and cardiovascular disease among older Latinos. However, less is known regarding the number of years and proportion of late life spent with self-reported cognitive impairment among this rapidly aging group and whether cognitive impairment life expectancy outcomes vary across U.S. Latino subgroups by country of origin. To fill this gap, our investigation uses data from the National Health Interview Survey (1997-2015) to estimate Sullivan-based life tables of cognitively normal and cognitively impaired life expectancies for adults 50 years and older. Results indicate significant heterogeneity among Latinos, with island-born Puerto Rican women spending the most years, and foreign-born Cuban men the fewest years lived with self-reported cognitive impairment.

